# HumTouch: Localization of Touch on Semi-Conductive Surfaces by Sensing Human Body Antenna Signal

**DOI:** 10.3390/s21030859

**Published:** 2021-01-28

**Authors:** Tzu Hsuan Hsia, Shogo Okamoto, Yasuhiro Akiyama, Yoji Yamada

**Affiliations:** Graduate School of Engineering, Nagoya University, Nagoya 464-8603, Japan; hsia.tzu-hsuan@i.mbox.nagoya-u.ac.jp (T.H.H.); yasuhiro.akiyama@mae.nagoya-u.ac.jp (Y.A.); yoji.yamada@mae.nagoya-u.ac.jp (Y.Y.)

**Keywords:** human touch sensing, human antenna, flexible touch sensing surfaces

## Abstract

HumTouch is a touch sensing technology utilizing the environmental electromagnetic wave. The method can be realized using conductive and semi-conductive materials by simply attaching electrodes to the object’s surface. In this study, we compared three methods for localizing a touch on $20\times 16\;{\mathrm{cm}}^{2}$ and $40\times 36\;{\mathrm{cm}}^{2}$ papers, on which four or eight electrodes were attached to record the voltages leaked from the human fingertip. The number and positions of the electrodes and the data processing of the voltages differed according to the localization methods. By constructing a kernel regression analysis model between the electrode outputs and the actual physical locations, the touched locations were estimated. Each of the three methods was tested via leave-one-out cross validation. Out of the three methods discussed, two exhibited superior performances in terms of the estimation errors. Of these two methods, one simply uses the voltages recorded by the four electrodes attached on the middle of paper edges as inputs to the regression system. The other uses differential outputs of electrode pairs as the inputs. The smallest mean location errors were 0.31 cm on $20\times 16\;{\mathrm{cm}}^{2}$ paper and 0.27 cm on $40\times 36\;{\mathrm{cm}}^{2}$ paper, which are smaller than the size of a fingertip.

## 1. Introduction

Touch sensing techniques are widely used in consumer electronics such as smartphones and laptop computers. These touch sensing techniques provide a more intuitive way to interact with the computer interface. Capacity sensing methods are a common technology used in these commercial devices [1,2,3]. These sensing methods use a specialized surface that is constructed using multiple layers of different materials. This method has been developed for years and is considerably mature and reliable. However, the number of studies focusing on transforming daily objects into touch-sensitive objects has been increasing [4,5,6,7,8,9,10,11,12,13,14,15,16,17,18,19,20]. Acoustic waves are used for localizing touch on rigid planes [4,5]. The disturbance source, i.e., finger, is located on a vibrating surface. This method requires transmitters to generate the acoustic wave and receivers to record the vibration of the surface. For flexible objects, the resistance and capacitance of materials, e.g., conductive fabrics and conductive rubber, are used for touch sensing [6,7,8,9,10]. When the surface deforms, the electrical properties of such materials change accordingly. Therefore, these materials are often used as pressure sensors and flexion sensors. Cameras are also used for touch localization [11,12,13,14,15]. This optical method does not depend on the surface material but is limited owing to the issue of occlusion. Electric tomography is used for conductive materials or insulative objects with conductive coating [16,17,18,19]. This method uses a relatively large number of electrodes. The current from one electrode to another changes with the conductive state of the surface. These methods [4,5,6,7,8,9,10,11,12,13,16,17,18,19,20,21] can potentially make daily objects, such as furniture and toys, touch-sensitive.

**Figure 1:**
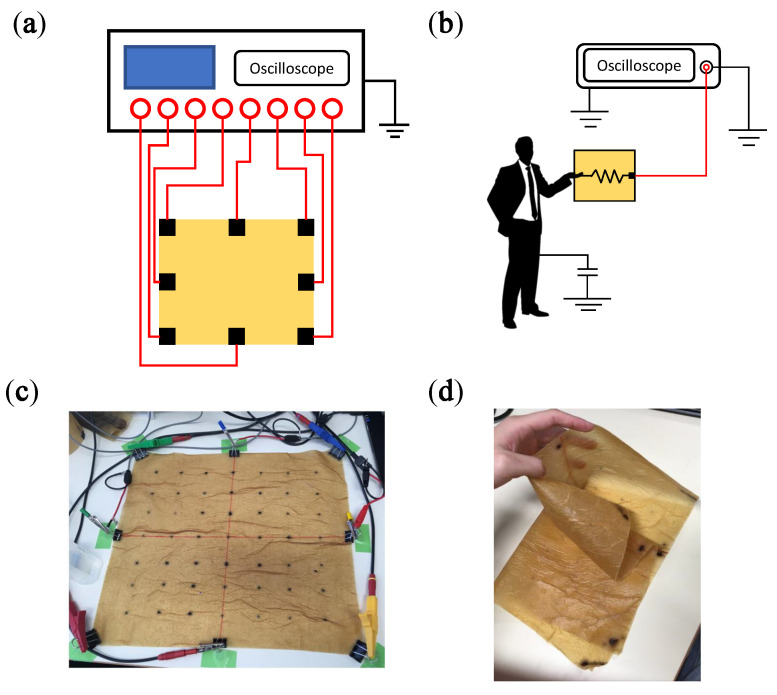
(**a**) Eight electrodes were attached to a semi-conductive paper. (**b**) A circuit was formed when a human touched the paper. (**c**) Photo of the paper ($40\times 36\;{\mathrm{cm}}^{2}$) with the electrodes attached. (**d**) The paper was dry, flexible, and durable.

In this study, we focus on a passive sensing method named HumTouch. Unlike the prominent methods that require surface activation, HumTouch utilizes environmental noise. Nowadays, AC power lines are widespread in our surroundings that generate 50/60 Hz electromagnetic waves. Further, the human body contains minerals and other conductive materials that react to the electromagnetic waves, inducing currents on the skin. These currents can be used for gesture recognition and human localization in a 3D space [22,23,24]. When a finger touches a conductive or semi-conductive material, the current leaks from the human body into the material surface. The voltage of this current can be detected via electrodes attached on the material, as shown in Figure 1a,b. Note that the positive electrodes are attached to the material surface, while the negative ones are grounded. Further, the human body is virtually coupled to the ground. In our pervious works, the application of HumTouch was validated for the development of a keyboard [25]. This method can be used with natural conductive materials such as granite [26,27]. For insulative materials, a semi-conductive hydrogel-paint for application to a 2D or 3D surface is available [28,29,30]. HumTouch does not require surface activation, which renders it different from other touch sensing methods. Hence, it can be potentially used with large-area surfaces and objects with limited volumes.

In our previous study, touch localization was achieved by constructing a kernel regression model with preprocessed voltages recorded at the electrodes [29,30]. The mean estimated errors in our former studies were 0.88 cm on a $16\times 19\;{\mathrm{cm}}^{2}$ paper and 0.22 cm on a 30-cm-long cylinder with the dimeter of 3 cm. These results motivated us to extend our research and investigate the preprocess methods for the collected voltages and the number and locations of electrodes. Herein, we demonstrate three different localization methods that use multiple electrodes and kernel regression analyses. The three methods are different in terms of the preprocessing of recorded voltages. One method does not apply any preprocessing. For the other two methods, linear combinations of the voltages are used for the input to the kernel regression analysis. Each of the three methods is tested on both $20\times 18\;{\mathrm{cm}}^{2}$ and $40\times 36\;{\mathrm{cm}}^{2}$ papers. Finally, we determine the best localization method for 2D HumTouch surfaces.

## 2. Materials and Methods

We painted a semi-conductive hydrogel on two different-sized wiping papers (20×18 cm and 40×36 cm, Kimtowel, Nippon Paper Crecia, Tokyo, Japan). The hydrogel [28] was prepared using the following constituents: 15 g of polyvinyl alcohol, 300 mL of ultrapure water, 75 mL polyethylene glycol 400, and 37.5 mL Glutaraldehyde. The solution was stirred for approximately 3 h at 90 °C. Then, the wiping papers were dried at room temperature for a week. After being dried, the papers remained flexible, and thus the strength does not apparently change. Nevertheless, we did not measure the exact change in the strength and stiffness after painting the hydrogel.

To measure the current, we attached eight electrodes to the paper as shown in Figure 1. An oscilloscope (HS6DIFF, TiePie, Netherlands; sampling frequency: 500 kHz) was used to record the voltage at each electrode. For each channel of the oscilloscope, one electrode was connected to the paper, and the other electrode was grounded.

**Figure 2:**
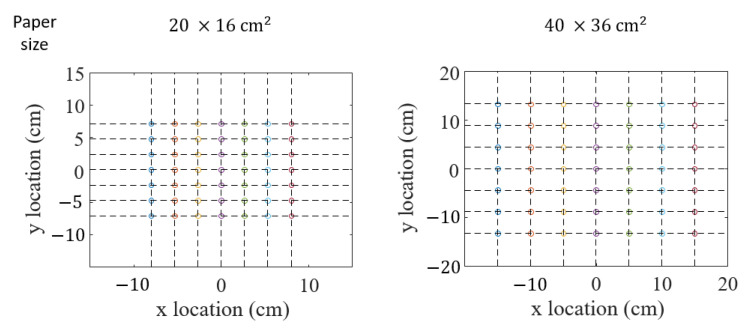
Points marked on the 20×16 and $40\times 36\;{\mathrm{cm}}^{2}$ paper.

**Figure 3:**
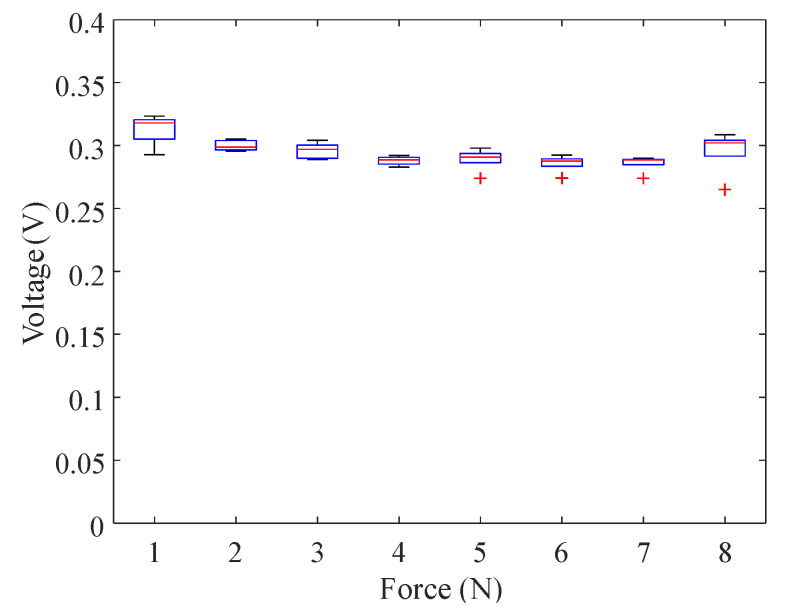
Voltages recorded at an electrode for different contact forces. The distance between the electrode and touched location was 12.8 cm. Five voltage values were recorded for each force.

We marked 7×7 points (49 total points) on both papers with a marker pen. As Figure 2 shows, the intervals between each point were 2.5 cm and 2.25 cm for the smaller paper and 5 cm and 4.5 cm for the larger paper. The papers were placed on a non-conductive wooden table inside a regular office room. A participant was asked to touch each of the 49 points with his bare index finger. The contact force was not fixed because the voltage barely changed with the contact force as shown in Figure 3, where the point that was 12.8 cm away from an electrode on a paper was repeatedly touched with the normal forces varying 1–8 N. The contact period for each point was approximately 1 s. This procedure was repeated seven times (seven data sets).

## 3. Localization Using Kernel Regression Model

### Kernel Regression Analysis

We applied kernel regression analysis as the model structure for our observations is unknown; however, the voltage at an electrode nonlinearly changes with the distance between the finger and electrode. The independent observations for learning the model are $\left(x_{1},y_{1}\right),\ldots ,\left(x_{j},y_{j}\right),\ldots ,\left(x_{n},y_{n}\right)$ where $x_{j}$ is the input vector, $y_{j}$ is the output scalar for the *j*th sample and *n* is the number of samples for learning. When the input vector is x, the estimated output $\tilde{y}$ is defined as

(1) $$\begin{array}{c} \tilde{y}=\sum_{j=1}^{n}\alpha_{j}k\left(x_{j},x\right) \end{array}$$

where $k(x_{j},x)$ is a kernel function. Coefficient $\alpha_{j}$ is a component of vector $\alpha ={\left(\alpha_{1},\ldots ,\alpha_{j},\ldots ,\alpha_{n}\right)}^{T}$, which is determined using

(2) $$\begin{array}{c} \alpha ={\left(K+\lambda I\right)}^{-1}\left[\begin{array}{c} y_{1}\\ \vdots \\ y_{j}\\ \vdots \\ y_{n} \end{array}\right]. \end{array}$$

Matrix $K\in R^{n\times n}$ is formed by $k\left(x_{i},x_{j}\right)\left(i,j=1,2,\ldots ,n\right)$, λ is the regularization value, and I is the identity matrix of n×n.

In our study, the location for each point was defined by the Cartesian coordinate system. We applied the kernel regression for each axis. Therefore, for the input vector x, the estimated two-dimensional location ${\left({\tilde{l}}_{x},{\tilde{l}}_{y}\right)}^{T}$ for each point can be written as

(3) $$\left[\begin{array}{c} {\tilde{l}}_{x}\\ {\tilde{l}}_{y} \end{array}\right]=\left[\begin{array}{c} \sum_{j=1}^{n}\alpha_{xj}k\left(x_{j},x\right)\\ \sum_{j=1}^{n}\alpha_{yj}k\left(x_{j},x\right) \end{array}\right]$$

where the coefficients $\alpha_{xj}$ and $\alpha_{yj}$ are the *j*th components of vectors $\alpha_{x}$ and $\alpha_{y}$, which are computed by

(4) $$\begin{array}{c} \alpha_{x}={\left(K+\lambda I\right)}^{-1}l_{x} \end{array}$$

(5) $$\begin{array}{c} \alpha_{y}={\left(K+\lambda I\right)}^{-1}l_{y}. \end{array}$$

Here, $l_{x}={\left(l_{x1},l_{x2},\ldots ,l_{xn}\right)}^{T}$ and $l_{y}={\left(l_{y1},l_{y2},\ldots ,l_{yn}\right)}^{T}$ are the actual x and y locations for the touched points. The kernel function used in our study is the Gaussian kernel, which is

(6) $$\begin{array}{c} k\left(x_{j},x\right)=\exp(-|x_{j}-x{|}^{2}) \end{array}$$

where |.| is the L2 norm. The regularization value λ was selected from 1 to $10^{-3}$ such that the smallest estimation error on average was achieved.

## 4. Three Voltage Processing Methods for Regression Model

### 4.1. Data Prepossessing Common for the Three Methods

A moving average filter with a span of five, i.e., a window size of 0.01 ms for a 500 kHZ sampling frequency of the oscilloscope, was used to smooth the signals recorded by the electrodes. The maximum voltage recorded by channel *i* during 1 s is $v_{i}$ and is used for the localization methods introduced in Section 4.2, Section 4.3 and Section 4.4.

### 4.2. Method 1: Simple Regression

**Figure 4:**
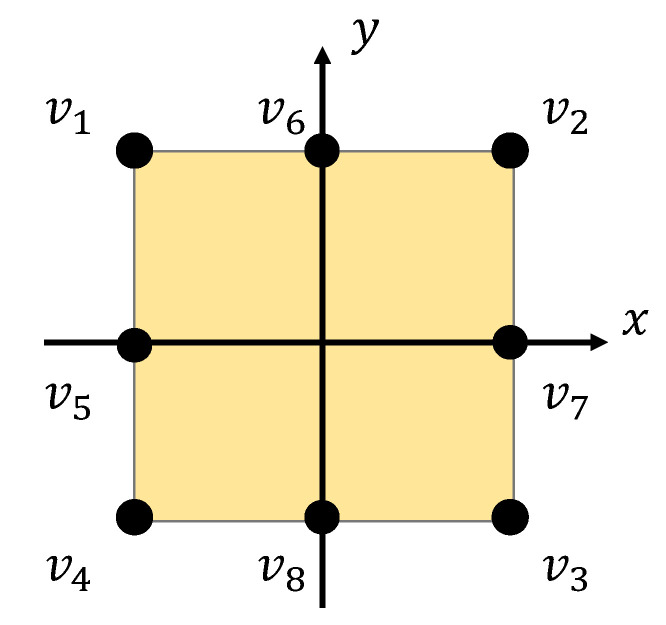
Loci of electrodes on the paper.

In this method, we used the voltage preprocessed in Section 4.1 as the input for the regression model. We constructed three slightly different regression models using this method. One model used the data collected by eight electrodes as the input. Therefore, the input was $x_{j}={\left(v_{1j},v_{2j},\ldots ,v_{8j}\right)}^{T}$, where $v_{ij}$ is the voltage output from the *i*th channel of sample *j*. Another model used the voltages recorded on the four corners $x_{j}=\left(v_{1j},v_{2j},v_{3j},v_{4j}\right)$ as the input, and the other used those on the middle of each edge $x_{j}=\left(v_{5_{j}},v_{6j},v_{7j},v_{8j}\right)$. The channel numbers and positions are shown in Figure 4. The model using eight channels is expected to be more accurate than the models using four channels.

### 4.3. Method 2: Regression Using Voltage Ratio

In our previous study [29], four electrodes were attached to the corners of a square paper. The localization was conducted via kernel regression using the voltage ratio defined by $x={\left(p_{x},p_{y}\right)}^{T}$:

(7) $$x_{j}=\left[\begin{array}{c} p_{xj}\\ p_{yj} \end{array}\right]=\left[\begin{array}{c} \frac{-v_{1j}+v_{2j}+v_{3j}-v_{4j}}{v_{1j}+v_{2j}+v_{3j}+v_{4j}}\\ \frac{v_{1j}+v_{2j}-v_{3j}-v_{4j}}{v_{1j}+v_{2j}+v_{3j}+v_{4j}} \end{array}\right].$$

These voltage ratios represent the touched location if the relationship between the channel output and distance between the electrode and fingertip is linear. However, the relationship is non-linear in reality, and we corrected this non-linearity by applying kernel regression analysis in [29].

In this study, we increased the number of channels to eight. Hence, we added two more parameters $p_{x2}$ and $p_{y2}$ using the voltage records at the middle of edges, i.e., $v_{5},v_{6},v_{7}$ and $v_{8}$. The input vector for sample *j* was $x_{j}={\left(p_{x1j},p_{y1j},p_{x2j},p_{y2j}\right)}^{T}$, where

(8) $$x_{j}=\left[\begin{array}{c} p_{x1j}\\ p_{y1j}\\ p_{x2j}\\ p_{y2j} \end{array}\right]=\left[\begin{array}{c} \frac{-v_{1j}+v_{2j}+v_{3j}-v_{4j}}{v_{1j}+v_{2j}+v_{3j}+v_{4j}}\\ \frac{v_{1j}+v_{2j}-v_{3j}-v_{4j}}{v_{1j}+v_{2j}+v_{3j}+v_{4j}}\\ \frac{-v_{5j}+v_{6j}+v_{7j}-v_{8j}}{v_{5j}+v_{6j}+v_{7j}+v_{8j}}\\ \frac{v_{5j}+v_{6j}-v_{7j}-v_{8j}}{v_{5j}+v_{6j}+v_{7j}+v_{8j}} \end{array}\right].$$

We expected the regression model built using the voltage ratio to be more accurate than the simple regression method because the input was related to the coordinates on the paper for the regression analysis.

### 4.4. Method 3: Regression Using Differential Output of Electrode Pair

We propose another method that utilizes the difference of preprocessed voltages between two channels as the input for regression models. The concept of this method is that although the magnitude of the voltage may be different among trials owing to environmental differences even if the same position is touched on the paper, the difference between the two channels’ outputs may be same among trails. Hence, the use of two channels’ difference is expected to lead to a smaller estimation error than the simple regression method. We used three different arrangements of the electrodes. One used the voltages measured at corners ($v_{1},\ldots ,v_{4}$). The input for sample *j* was defined as

(9) $$x_{j}=\left[\begin{array}{c} c_{1j}\\ c_{2j}\\ c_{3j}\\ c_{4j}\\ c_{5j}\\ c_{6j} \end{array}\right]=\left[\begin{array}{c} v_{1j}-v_{2j}\\ v_{1j}-v_{3j}\\ v_{1j}-v_{4j}\\ v_{2j}-v_{3j}\\ v_{2j}-v_{4j}\\ v_{3j}-v_{4j} \end{array}\right].$$

Another arrangement used the voltages measured at the middle of edges ($v_{5},\ldots ,v_{8}$). In this case, the input for sample *j* was

(10) $$x_{j}=\left[\begin{array}{c} d_{1j}\\ d_{2j}\\ d_{3j}\\ d_{4j}\\ d_{5j}\\ d_{6j} \end{array}\right]=\left[\begin{array}{c} v_{5j}-v_{6j}\\ v_{5j}-v_{7j}\\ v_{5j}-v_{8j}\\ v_{6j}-v_{7j}\\ v_{6j}-v_{8j}\\ v_{7j}-v_{8j} \end{array}\right].$$

Furthermore, we tested the input computed by the eight electrodes. The input for sample *j* contained 28 elements (${}_{8}C_{2}$) and was defined as

(11) $$x_{j}=\left[\begin{array}{c} e_{1j}\\ e_{2j}\\ \vdots \\ e_{28j} \end{array}\right]=\left[\begin{array}{c} v_{1j}-v_{2j}\\ v_{1j}-v_{3j}\\ \vdots \\ v_{7j}-v_{8j} \end{array}\right].$$

## 5. Results

### 5.1. Performance Evaluation Method

We applied the leave-one-out cross validation method to investigate the estimation error. Six of the seven data sets (294 out of 343 points) were used to build the regression model and the remaining data set (49 out of 343 points) was used to test the estimation performance. This procedure was conducted for every data set (repeated seven times), and then, the means and standard deviations of the errors were calculated.

### 5.2. Simple Regression

**Figure 5:**
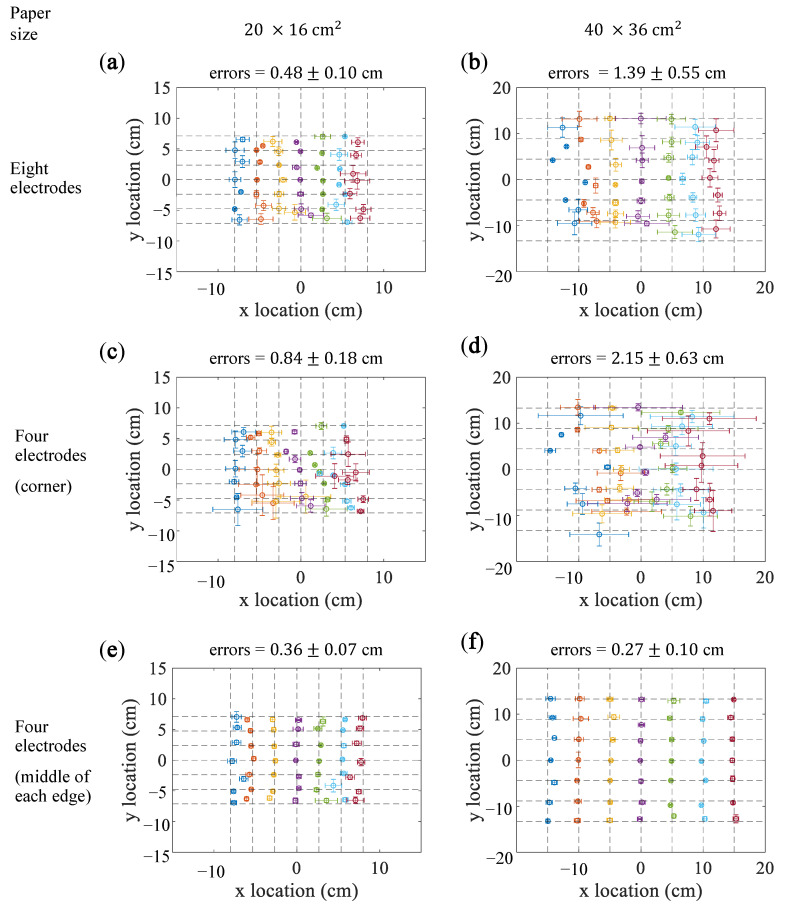
Results of the simple regression methods. (**a**,**c**,**e**) show the results for the $20\times 16\;{\mathrm{cm}}^{2}$ paper. (**b**,**d**,**f**) show the results for the $40\times 36\;{\mathrm{cm}}^{2}$ paper. (**a**,**b**) show the results obtained using eight electrodes. (**c**,**d**) shows the results obtained using four electrodes placed in the corners of the paper. (**e**,**f**) show the results obtained using four electrodes placed in the middle of the edges of the paper. The points denote the mean estimated locations for the intersection points, and the error bars denote the standard deviations.

The points shown in Figure 5 are the mean estimated locations and the standard deviations for individual points. The intersections of the grids denote the actually touched locations. Figure 5a,b are the results when the eight electrodes were used for localization. The estimation error was 0.48±0.10 cm for the $20\times 16\;{\mathrm{cm}}^{2}$ paper and 1.39±0.55 cm for the $40\times 16\;{\mathrm{cm}}^{2}$ paper. For both papers, the estimation error and the standard deviation for the points near the edges were larger than those of the points near the center of the paper.

Figure 5c,d present the results when the four electrodes were placed on the corners ($x=(v_{1},\ldots ,v_{4})$). The mean estimation errors were 0.84±0.18 cm and 2.15±0.63 cm for the $20\times 16\;{\mathrm{cm}}^{2}$ and $40\times 36\;{\mathrm{cm}}^{2}$ paper, respectively.

Figure 5e,f illustrate the results when the electrodes were placed on the middle of edges ($x=(v_{5},\ldots ,v_{8})$). The mean estimation errors were 0.36±0.07 cm and 0.27±0.10 cm for the 20×16 cm and $40\times 36\;{\mathrm{cm}}^{2}$ paper, respectively. For the $20\times 16\;{\mathrm{cm}}^{2}$ paper, the estimation error was larger for the points close to the edges than those near the center of the paper.

### 5.3. Voltage Ratios

**Figure 6:**
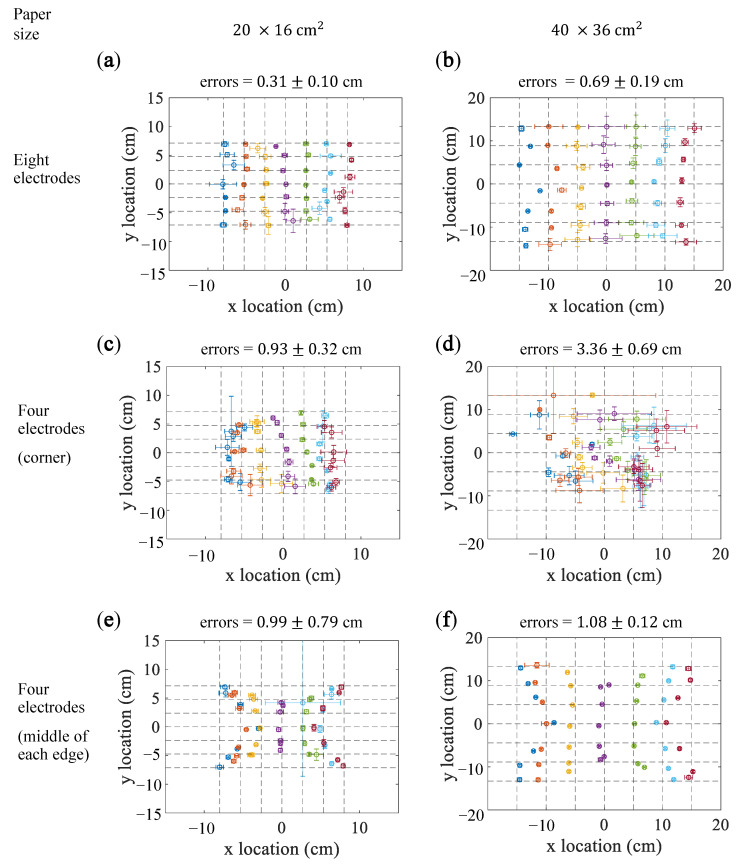
Results of the voltage ratio method obtained using four or eight electrodes. (**a**,**c**,**e**) show the results for the $20\times 16\;{\mathrm{cm}}^{2}$ paper. (**b**,**d**,**f**) show the results for the $40\times 36\;{\mathrm{cm}}^{2}$ paper. (**a**,**b**) show the results obtained using eight electrodes. (**c**,**d**) show the results obtained using four electrodes placed in the corners of the paper. (**e**,**f**) show the results obtained using four electrodes placed in the middle of the edges of the paper. The points denote the mean estimated locations for the intersection points, and the error bars denote the standard deviations.

Figure 6a,b present the results for the method using voltage ratios calculated by the eight electrodes. The estimation errors were 0.31±0.09 cm for the smaller paper and 0.69±0.19 cm for the lager paper.

Figure 6c,d illustrate the results for the method using the four electrodes on corners. The mean estimation errors were 0.93±0.32 cm for the 20×16 ${\mathrm{cm}}^{2}$ paper and 3.26±0.69 cm for the $40\times 36\;{\mathrm{cm}}^{2}$ paper. For the 20×16 ${\mathrm{cm}}^{2}$ paper, the estimation errors for the points near edges are larger than those near the center of the paper. The estimation errors for the 40×36 ${\mathrm{cm}}^{2}$ paper were evidently larger than those for the smaller paper.

Figure 6e,f present the results for the method using four electrodes placed on the middle of edges. The estimation errors were 0.99±0.79 cm for the smaller paper and 1.08±0.12 cm for the lager paper.

### 5.4. Differential Output of Electrode Pair

**Figure 7:**
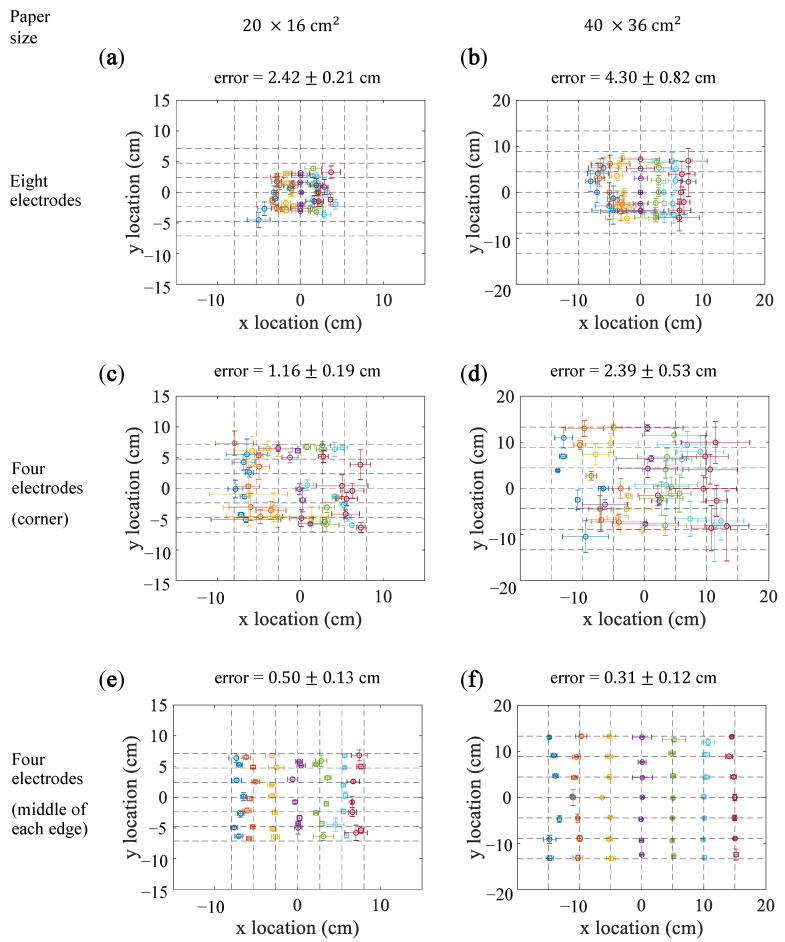
Results of the differential output methods. (**a**,**c**,**e**) show the results for the $20\times 16\;{\mathrm{cm}}^{2}$ paper. (**b**,**d**,**f**) show the results for the $40\times 36\;{\mathrm{cm}}^{2}$ paper. (**a**,**b**) show the results obtained using eight electrodes. (**c**,**d**) show the results obtained using four electrodes placed in the corners of the paper. (**e**,**f**) show the results obtained using four electrodes placed in the middle of the edges of the paper. The points denote the mean estimated locations for the intersection points, and the error bars denote the standard deviations.

Figure 7 shows the results for the differential output methods. Figure 7a,b show the estimation based on the corner electrodes, for which the localization of the touched points was failed. The estimation errors were 2.42±0.21 on the small paper and 4.30±0.82 on the large paper. As shown in Figure 7c,d, the estimation errors for the method using the four electrodes at corners for the $20\times 16\;{\mathrm{cm}}^{2}$ and $40\times 36\;{\mathrm{cm}}^{2}$ papers were 1.16±0.19 cm and 2.39±0.53 cm, respectively. As shown in Figure 7e,f, for the method using the four electrodes on the middle of edges, the estimation errors were 0.50±0.13 cm and 0.31±0.12 cm for the $20\times 16\;{\mathrm{cm}}^{2}$ and the $40\times 36\;{\mathrm{cm}}^{2}$ paper, respectively.

### 5.5. Summary and Comparison of Three Methods

Table 1 shows the mean estimation error and standard deviation for each method. The simple regression method using the electrodes on the middle of edges, voltage ratio method using eight electrodes, and differential output method using the electrodes on the middle of edges exhibited relatively small localization errors.

We tested the statistical differences in the mean errors between the method with the smallest error and the others for each type of the paper, and the results are summarized in Table 2. For the small paper, the method based on the voltage ratio of the eight electrodes, exhibited smaller errors than the other methods except for the simple regression method and differential output method using the electrodes on the middle of edges. In contrast, the simple regression method using the middle of edges showed the smallest error for the large paper. The error was smaller than those of the other methods expect for the simple regression method using eight electrodes, the voltage ratio method using eight electrodes, the voltage ratio method using electrodes on the middle of edges, and the differential output method using the electrodes on the middle of edges.

## 6. Discussion

The following three methods resulted in small localization errors: the simple regression method using the middle of the edges of the paper; the voltage ratio method using eight electrodes; and the differential output method using the middle of the edges of the paper. These methods did not exhibit statistical differences. The mean localization errors of three methods were smaller than a fingertip. These errors can be compared with those obtained by other touch sensing methods such as an electric tomography method with maximum mean localization errors of 10 mm and a method using an inertial measurement unit sensor with a maximum position error of 2.5 mm [19,31]. Among all the localization methods, the simple regression method using the middle of the edges of the paper is better than the other methods because of the low number of electrodes and mean localization errors.

**Figure 8:**
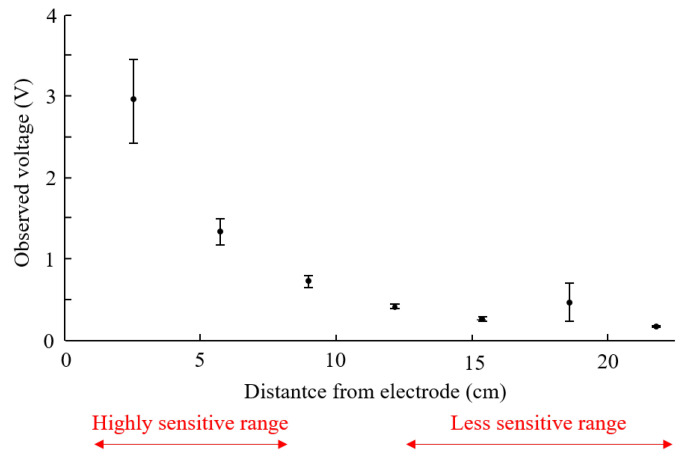
Voltages measured at an electrode for a finger placed at different distances. The points denote the mean voltages for the seven measurements obtained, and the error bars denote the standard deviation.

The points near the edges incur larger estimation errors and standard deviations than those near the center of the papers. This can be explained based on the relationship between the measured voltages and finger-electrode distances, shown in Figure 8. The points shown in the figure denote the mean voltages measured at different distances from an electrode, and the error bars denote the standard deviation. For the points near the edges, the distance between the finger and electrode is either laid on highly sensitive distances or less sensitive distances. In the highly sensitive range, the voltage considerably varies when the location touched by the finger slightly varies, causing large standard deviations. In contrast, in the less sensitive range, the voltage does not evidently vary even when the location touched by the finger substantially varies. Therefore, the estimations for the points near edges are inaccurate.

In general, the mean estimation error for the $40\times 36\;{\mathrm{cm}}^{2}$ paper was larger than that for the $20\times 16\;{\mathrm{cm}}^{2}$ paper. The mean estimation errors for the $40\times 36\;{\mathrm{cm}}^{2}$ and $20\times 16\;{\mathrm{cm}}^{2}$ papers were 1.77 cm and 0.89 cm, respectively. The dimensions of the larger paper were nearly twice those of the smaller paper. This indicates that the lager are the papers, the greater are the localization errors. This could be because, for the larger paper, the distances between the touched locations and electrodes are large, and the sensitivity of the output voltage is low.

For the differential output method, the estimation errors obtained when using four electrodes are smaller than those obtained using eight electrodes, which disagrees with our initial expectation. This could potentially be because of the overlearning of the regression model. For the method using eight electrodes, the number of explanatory variables was 28 (${}_{8}C_{2}$), while that for the methods using four electrodes was six. The model established based on the differential output method using eight electrodes might have been extremely specific to the data used for learning and not for general purposes.

HumTouch utilizes the environmental noise that cannot be easily replicated; thus, the localization results could depend on the participant and environment. In the future, the regression models constructed based on different participants and environments need to be compared. Further, although we used the Gaussian kernel functions, these functions were not optimized. Better kernel functions that could help obtain more accurate results can be determined. An equivalent circuit model of HumTouch is yet to be established and studied. Once the model is established, a computational optimization of electrode positions and numbers can be realized.

## 7. Conclusions

HumTouch is a passive sensing technology that can be used for semi-conductive materials; furthermore, this technology does not require surface activation or specialized surface structures. In this study, the localization of touch via the HumTouch technology was realized on two different-sized papers ($20\times 16\;{\mathrm{cm}}^{2}$ and $40\times 36\;{\mathrm{cm}}^{2}$). Three different localization methods were tested on both papers for different electrode positions. The simple regression method using electrodes at the middle of the edges of the paper, the voltage ratio method using eight electrodes, and the differential output method using electrodes at the middle of the edges of the paper exhibited smaller localization errors than the other methods. The smallest errors for the $20\times 16\;{\mathrm{cm}}^{2}$ and $40\times 36\;{\mathrm{cm}}^{2}$ paper were 0.31±0.09 cm and 0.27±0.10 cm, respectively. These errors are smaller than a fingertip. The simple regression method using four electrodes at the middle of the four edges of the paper is the most commonly applied localization method since this method requires only four electrodes and incurs small errors. In the future, we will seek applications suitable for HumTouch.

## Figures and Tables

**Table 1:** Mean estimation error and standard deviation (cm) for each method.

Localization Method	Number of Electrodes	20×16 cm${}^{2}$ Paper	$40\times 36\;{\mathrm{cm}}^{2}$ Paper
Simple regression	8	0.48±0.10	1.39±0.55
Simple regression (corner)	4	0.84±0.18	2.15±0.63
Simple regression (middle of edges)	4	0.36±0.07	0.27±0.10
Voltage ratio	8	0.31±0.09	0.69±0.19
Voltage ratio (corner)	4	0.93±0.32	3.36±0.69
Voltage ratio (middle of edges)	4	0.99±0.79	1.08±0.12
Differential output	8	2.42±0.21	4.30±0.82
Differential output (corner)	4	1.16±0.19	2.39±0.53
Differential output (middle of edges)	4	0.50±0.13	0.31±0.12

**Table 2:** *t* and *p* values for each paper when the method with the smallest error and the others are statistically compared by *t*-tests without correction of *p* values. The degrees of freedom were six for all tests.

Localization Method	$20\times 16\;{\mathrm{cm}}^{2}$ Paper		$40\times 36\;{\mathrm{cm}}^{2}$ Paper	
	t	p	t	p
Simple regression with 8 electrodes	2.97	0.025	1.83	0.12
Simple regression (corner)	2.78	0.032	2.78	0.032
Simple regression (middle of edges)	0.30	0.78	N/A	N/A
Voltage ratio with 8 electrodes	N/A	N/A	0.72	0.50
Voltage ratio with 4 electrodes (corner)	8.20	0.0002	3.82	0.009
Voltage ratio with 4 electrodes (middle of edges)	3.52	0.013	−0.84	0.43
Differential output with 8 electrodes	9.25	0.00009	3.78	0.0092
Differential output (corner)	3.87	0.008	2.48	0.036
Differential output (middle of edges)	1.72	0.14	1.064	0.33

## Data Availability

Data can be requested by direct email contacts to the author.
